# A Neurodiversity-Oriented Approach to Address Autism Wandering as a “Problem Behavior” in Pediatrics

**DOI:** 10.7759/cureus.40862

**Published:** 2023-06-23

**Authors:** Emily Hotez, Morénike Giwa Onaiwu

**Affiliations:** 1 General Internal Medicine, David Geffen School of Medicine at University of California Los Angeles, Los Angeles, USA; 2 General Practice, University of East Anglia, Norwich, GBR

**Keywords:** pediatricians, neurodiversity, children, autism, wandering

## Abstract

Pediatricians are frequently tasked with addressing autism “problem behaviors,” including wandering, defined as leaving the safety of a responsible person’s care or a safe area (alternatively referred to as elopement). In the following commentary, we - as autism researchers and individuals with lived experience - discuss the prevalence and public health consequences of wandering. We conceptualize wandering in the context of “problem behaviors” for autistic individuals and describe the current state of the evidence on wandering prevention and intervention. We emphasize that pediatricians have a unique opportunity to optimize their efforts to address wandering - as well as related “problem behaviors” - utilizing a neurodiversity orientation. This will allow them to enact approaches that address the potential upstream mechanisms underlying wandering to make these efforts more effective and provide critical assistance to families. In this manuscript, we provide recommendations to pediatricians to more effectively address the mechanisms underlying and exacerbating these challenges to improve the health, well-being, and quality of life of autistic children and their families. In particular, we recommend that pediatricians focus efforts toward 1) addressing the link between chronic stress and "problem behaviors"; 2) engaging individuals, caregivers, and families as experts in their health and development; and 3) collaborating with the systems and sectors relevant to autistic individuals and their families.

## Editorial

Background

Approximately one- to two-thirds of autistic children are prone to wandering, defined as leaving a responsible person’s care or a safe area (alternatively referred to as elopement) [1,2]. Wandering often occurs in the form of the child running away at school or leaving the house when the family is not looking [3]. Wandering can also occur in adolescents and adults, particularly among those with co-occurring intellectual disability [4,5]. Specific groups may be at heightened risk for wandering, including autistic children ages five to nine and those with certain communication challenges [1].

Wandering is a significant public health concern. The National Autism Association collected five years of data on missing person cases involving autistic children in the United States. Of the 808 cases identified in this analysis, 17% resulted in death, 13% required medical attention, and 38% carried a higher risk of bodily harm [1]. Accidental drowning accounted for 71% of lethal outcomes, followed by 18% caused by traffic injuries [1]. Police intervention was utilized in over a third of the cases [1]. Over 60% of parents of autistic individuals cite wandering as a direct source of increased stress and reduced quality of life [4]. Generally, estimates suggest that, each month, there are roughly 20 cases of autism wandering, and two to three of those cases result in death [6].

Current approaches to wandering

Wandering is typically conceptualized as one example of autism “problem” or “challenging” behaviors, along with self-injurious behavior, repetitive tendencies, and acute stress reactions, often perceived as “tantrums" [7]. Prevention approaches often take the form of alarms, installing locks on doors, blocking and restraint, teaching safety skills, and encouraging children to learn their phone number and address. As with other “problem behaviors,” intervention approaches typically focus on functional approaches (identifying and manipulating behaviors utilizing reinforcements or punishments) [8].

To be sure, existing preventive and functional approaches - comprehensively outlined in the American Academy of Pediatrics (AAP) Toolkit on Wandering [3] and stakeholder and federal workgroup recommendations [9,10] - are important for promoting safety. There is an important opportunity, however, for pediatricians to enact approaches that address the potential upstream mechanisms underlying wandering to make these efforts more effective and provide critical assistance to families.

A neurodiversity-oriented approach to wandering

In 2022, the Autism Intervention Research Network on Physical Health (AIR-P) published its first Pediatrics Supplement, which helped lay the foundation for a neurodiversity-oriented approach to care for autistic individuals. In this approach, the pediatric clinic-including the physical environment, staff demographics, clinical interactions, and office culture-is a) designed to be responsive to the diverse needs, experiences, and preferences of individuals across the life course and b) focused on promoting health, well-being, and thriving, rather than on trying to “cure” or “normalize” autistic individuals.

Address the Link Between Chronic Stress and “Problem Behaviors”

Across the life course, autistic individuals experience a variety of profound chronic stressors: persistent and cumulative stressful experiences, including social isolation, exclusion, and discrimination; experiences of abuse and trauma; and/or general lack of fit between individual and environmental factors [11-17]. These experiences begin in childhood [18]. There is a burgeoning evidence base suggesting that chronic stressors may lead to chronic high cortisol and psychological distress, which, in turn, lead to maladaptive coping behaviors in times of stress [11-17,19,20]. In addition, wandering often occurs when an individual experiences heightened duress, emotional or sensory over- or under-stimulation, and/or periods of transition or unexpected change [4,21,22].

During routine visits, pediatricians have the opportunity to ask families about the presence of stressors in children’s ecosystems and give them strategies to mitigate the effects of such stressors. There is a paucity of research on the trauma experiences of autistic individuals [23]. Pediatricians can, however, refer to the “practice pathway,” a tool designed to help pediatricians generate individualized treatment plans, taking into account psychosocial stressors, including abuse, victimization, and other environmental factors [24]. They can also apply inclusive language guidelines to ensure that their language during clinical encounters is non-stigmatizing and does not elicit additional stress (e.g., utilizing more descriptive examples of behaviors versus utilizing terminologies, such as "problem behavior") [25,26]. Generally, pediatricians can inquire about individual and family preferences and tailor interactions accordingly, particularly for those with multiple marginalized intersectional identities.

Engage Individuals, Caregivers, and Families as Experts in Their Health and Development

Despite the high incidence of wandering, only one-third of caregivers report receiving counseling on the issue from healthcare professionals [27]. Although the specific reasons are not well documented in the literature, this finding may be due to the lack of time, training, or other factors. Pediatricians can promote family and individual agency, self-determination, and self-advocacy by discussing wandering in routine visits. While pediatricians may be accustomed to discussing the presence of “problem behaviors,” there may be less discussion and provider confidence on the reasons for such behaviors. In working with families, pediatricians have the opportunity to empower caregivers to consider why wandering may occur. This can lead to more individualized and tailored guidance.

Conversations about wandering can continue over time and align with the developmental trajectory of the autistic individual. In practice, this entails ensuring that the pediatrician is directly interacting with the individual, rather than exclusively communicating with the caregiver over the course of development. Pediatricians can refer to the Academic Autistic Spectrum Partnership in Research and Education (AASPIRE) toolkit, which provides tools for both patients and providers to support patients’ agency in healthcare interactions and treatment plans [28].

Collaborate with the Systems and Systems and Sectors that are Relevant to Autistic Individuals and their Families

Wandering may occur across settings, including school, public outings, therapy settings, and home. Pediatricians have the opportunity to partner with caregivers to ensure that these settings are meeting the child’s needs. In particular, they can work with families to identify strategies for enhancing social inclusion and connectedness with peers and teachers. Pediatricians can also collaborate with educators, school psychologists, and other school-based or private mental health providers in their work with specific patients. They can work with schools to develop services and support, advocate for greater flexibility in teaching and learning practices, and promote attunement to diverse learning styles and preferences [29,30].

Pediatricians who are familiar with community-based services and resources may be well positioned to support families in securing other social resources beyond the school system. These resources can further mitigate chronic stressors, bolster inclusion, and promote self-advocacy [4]. Furthermore, pediatricians can leverage their roles as trusted, knowledgeable professionals to advocate for policy and societal changes that can help mitigate dangers posed by wandering. These changes may include innovative community integration efforts for autistic individuals, meaningful involvement of such individuals in the development of programs that address wandering, and combating racism and ableism in law enforcement interactions [31].

Applying this framework and drawing on contemporary research, we present recommendations and resources for pediatricians (see Table 1). All recommended efforts would ideally be proactive (i.e., occurring before “problem behaviors” occur) and developmentally based (i.e., aligned with developmental milestones), with the understanding that flexibility is needed depending upon unique characteristics and/or circumstances.

**Table 1:** Recommendations, research basis, and resources for pediatricians to address wandering and other “problem behaviors” These recommendations should supplement existing protocols and practices recommended by the American Academy of Pediatrics, particularly those related to safety and emergency preparedness [3].

Recommendation	Research basis	Resources
Address the link between chronic stress and “problem behaviors”	Autistic children experience pronounced chronic stressors. Chronic stressors may be the underlying mechanism to “problem behaviors.” Pediatricians have the opportunity to address chronic stressors in their autistic patients.	*Irritability and Problem Behavior in Autism Spectrum Disorder: A Practice Pathway for Pediatric Primary Care* (McGuire et al., 2016) [24]
Engage individuals, caregivers, and families as experts in their health and development	Individuals, caregivers, and families experience high distress and low resources and supports related to wandering. Pediatricians have the opportunity to engage them as experts to offer case-by-case strategies to address “problem behaviors."	Academic-Autistic Spectrum Partnership in Research and Education (AASPIRE)
Collaborate with the systems and sectors relevant to autistic individuals and their families	Pediatricians’ continuous coordination with families and other service systems make them well equipped to bolster supports for children in schools, where wandering often occurs. Wandering often results in police interventions.	*Linking the Medical and Educational Home to Support Children With Autism Spectrum Disorder: Practice Recommendations *(Shahidullah et al., 2018) [30]

Conclusion

As autism researchers and individuals with lived experience, it is clear from our vantage point that the adoption of these neurodiversity-oriented practices and resources will more effectively address the public health consequences of wandering and “problem behaviors” more broadly. We look forward to future efforts that will continue to address the potential upstream mechanisms underlying wandering and provide critical assistance to autistic individuals and their families.
